# Fabrication and Biological Activities of Plasmid DNA Gene Carrier Nanoparticles Based on Biodegradable l-Tyrosine Polyurethane

**DOI:** 10.3390/ph15010017

**Published:** 2021-12-24

**Authors:** Soo-Yong Park, Yang H. Yun, Bum-Joon Park, Hyung-Il Seo, Ildoo Chung

**Affiliations:** 1 Department of Polymer Science and Engineering, Pusan National University, Busan 46241, Korea; sooyong7466@gmail.com; 2 Department of Biomedical Engineering, College of Engineering, The University of Akron, Akron, OH 44325, USA; yy@uakron.edu; 3 Department of Molecular Biology, College of Natural Science, Pusan National University, Busan 46241, Korea; bjpark1219@pusan.ac.kr; 4 Department of Surgery, Biomedical Research Institute, Pusan National University Hospital, Busan 49241, Korea; seohi71@hanmail.net

**Keywords:** gene carrier, biodegradable, LTU, double emulsion, DNA-LPEI complex, transfection

## Abstract

Gene therapy is a suitable alternative to chemotherapy due to the complications of drug resistance and toxicity of drugs, and is also known to reduce the occurrence of cellular mutation through the use of gene carriers. In this study, gene carrier nanoparticles with minimal toxicity and high transfection efficiency were fabricated from a biocompatible and biodegradable polymer, l-tyrosine polyurethane (LTU), which was polymerized from presynthesized desaminotyrosyl tyrosine hexyl ester (DTH) and polyethylene glycol (PEG), by using double emulsion and solvent evaporation techniques, resulting in the formation of porous nanoparticles, and then used to evaluate their potential biological activities through molecular controlled release and transfection studies. To assess cellular uptake and transfection efficiency, two model drugs, fluorescently labeled bovine serum albumin (FITC-BSA) and plasmid DNA-linear polyethylenimine (LPEI) complex, were successfully encapsulated in nanoparticles, and their transfection properties and cytotoxicities were evaluated in LX2 as a normal cell and in HepG2 and MCF7 as cancer cells. The morphology and average diameter of the LTU nanoparticles were confirmed using light microscopy, transmission electron microscopy, and dynamic light scattering, while confocal microscopy was used to validate the cellular uptake of FITC-BSA-encapsulated LTU nanoparticles. Moreover, the successful cellular uptake of LTU nanoparticles encapsulated with pDNA-LPEI and the high transfection efficiency, confirmed by gel electrophoresis and X-gal assay transfection, indicated that LTU nanoparticles had excellent cell adsorption ability, facilitated gene encapsulation, and showed the sustained release tendency of genes through transfection experiments, with an optimal concentration ratio of pDNA and LPEI of 1:10. All the above characteristics are ideal for gene carriers designed to transport and release drugs into the cytoplasm, thus facilitating effective gene therapy.

## 1. Introduction

A number of attempts have been made to focus on the development of gene therapy to cure inheritable as well as congenital and acquired diseases [1,2,3,4]. However, it has been encumbered by stability problems, such as toxicity or host immune responses. The most pertinent solution for gene therapy is to develop carriers (vectors) because exposed DNA molecules cannot enter cells due to their large size and hydrophilic properties, which arise from negatively charged phosphate groups. It has been reported that non-viral gene delivery includes two types of common methods involving the use of nucleic acid complexed with cationic lipids (lipoplexes) or cationic polymers (polyplexes). The cationic polymer polyplexes have an appropriate size, which facilitates their uptake into cells through self-assembly, due to electrostatic attraction with nucleic acids [5,6,7,8,9].

l-tyrosine polyurethane (LTU) is a biodegradable polyurethane that is classified as a “pseudo” poly (amino acid), polymeric materials that are obtained from homo poly (amino acids) by the incorporation of hydrolytically degradable, non-peptide bonds along with enzymatically degradable peptide bonds within the polymer backbone. These materials have favorable processing, increased hydrolytic degradation, and adjustable swelling properties [10]. LTU is designed to undergo degradation through hydrolysis and enzymatic mechanisms, since the backbone of the polymer contains a peptide bond together with polyethylene glycol (PEG) as a soft segment macrodiol, which minimizes the material cytotoxicity of polyurethanes while simultaneously increasing their flexibility [11,12,13]. These properties of LTU make it promising for gene delivery applications that require the sustained release of DNA throughout the entire lifetime of the cell. In contemporary years, a number of our previous research studies have been applied to develop biodegradable nanoparticles based on aliphatic polyester, poly(amino acid), polyanhydride, and biopolymer and used to evaluate their biological activities and cytotoxicities as antitumoral and antibacterial agents, and as gene carriers [14,15,16].

We previously reported a study focusing on the therapeutic effect of microRNA-378a on liver fibrosis using LTU nanoparticles as a carrier to deliver microRNA-378a into an animal model. Furthermore, the results directly proved our hypotheses about the mechanism of liver fibrosis and provided a promising route for using micoRNA-378a as both a biomarker of liver pathogenesis and a therapeutic agent for liver disease [17].

The unique properties of nanomaterials, combined with material diversity and myriad design considerations, have all been used in the production of non-viral vectors and, thus, were selected in the present study. These nanoscale materials, comprising cationic polyplexes, lipoplexes, and liposomes, can together be categorized as biodegradable nanoparticles [18]. Ideally, the carriers used in non-viral gene therapy should be able to deliver the encapsulated genetic material to the targeted location in cells and into the nucleus of the cell without causing toxicity or eliciting an immune response. For efficient uptake into cells, non-viral delivery carriers must be of an appropriate size for endocytosis, following which they are degraded in the body to prevent toxicity due to carrier accumulation, and thus, they should be able to release the genetic material before the cell death in order to achieve a high delivery efficiency [15,19]. To improve the relatively low transfer efficiency compared to the commonly used viral vectors, plasmid DNA (pDNA)-linear polyethylenimine (LPEI) was used as a complex with DNA, and its surface was modified with PEG using the double emulsion method. Although pDNA-LPEI has enhanced cell adsorption capacities due to the cationic nature of LPEI, its high toxicity and low stability are major factors limiting its use as a gene carrier to the immune system. Considerable efforts have been made to overcome these problems, which include encapsulation by biocompatible polymers such as PEG and PLA, and the fabrication of stable nanoparticles in the blood circulation system [20,21,22]. Usually, hydrophilic surface nanoparticles have the disadvantage of being rapidly removed from the blood by the reticuloendothelial system (RES) in the liver or spleen cells. To overcome this disadvantage, surface modification with PEG, known as PEGylation, is an effective method that not only inhibits the growth of the bacteria but also aids the molecule in avoiding detection by the immune system, which is a characteristic of retroviral vectors [23,24,25].

Therefore, the present study focused on developing a highly stable non-viral and non-immunogenic gene delivery system that avoids large DNA sizes, in addition to having the potential for use in therapeutic applications. In this study, LTU nanoparticles (NPs) were synthesized using the water-in-oil-in-water (w/o/w) double emulsion method, and these consist of an inner water pocket with fluorescently labeled bovine serum albumin (FITC-BSA) or DNA-LPEI polyplex, a middle oil phase comprising biodegradable polymer LTU, and surface modifications with PEG. The double emulsion technique offers many interesting possibilities for the controlled release of encapsulated materials in internal droplets, and it has been studied as a release medium for various hydrophilic materials. Double emulsions could be formed by using two emulsifiers with opposite solubilities, water-soluble and oil-soluble [26,27]. The amphiphilic methoxy poly(ethylene glycol)-b-poly(l-lactide) (mPEG-PLA) block copolymer was also synthesized and used as a surfactant, and PEGylation was accomplished using an aqueous solution of 5% PVA for stabilization [28,29]. pDNA-PEI complexes (polyplex) have a high transfection efficiency both in vitro and in vivo because they are protonated by endosomal ATPase after degradation by lysozymes, and swelling due to osmotic pressure occurs owing to the influx of chloride ions [30,31]. This results in a high transfection efficiency due to physical destruction, which is termed the “proton sponge effect” [32]. Although PEI demonstrates competent levels of transfection efficiency, it has high cytotoxicity due to the presence of many amine groups [33,34]. Hence, LPEI (25 kDa) was prepared to minimize the cytotoxicity, where it existed in the inner pocket of our NPs, by forming a complex with the pDNA. It was expected that these LTU NPs would possess minimal material toxicity and immunogenicity, reduce accumulation toxicity by virtue of their biodegradability and biocompatibility properties, and demonstrate high transfection efficiency owing to the use of LPEI and surface modification with PEG.

## 2. Results and Discussion

### 2.1. Synthesis and Characterization of LTU and mPEG-PLA

**Figure 1:**
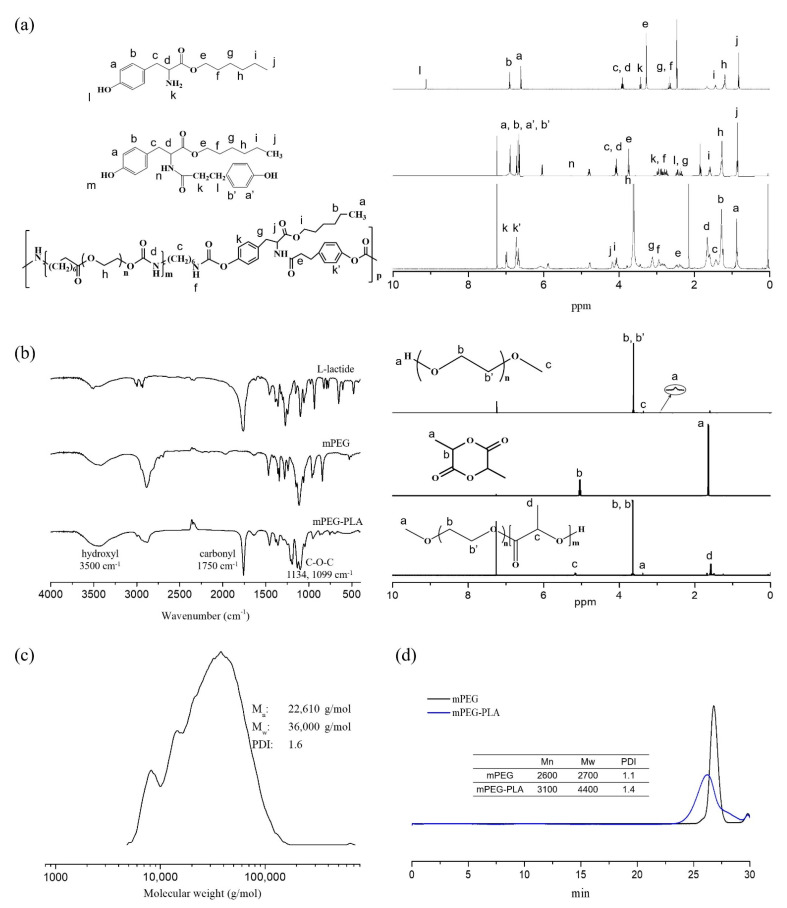
(**a**) ^1^H-NMR spectra of TH, DTH, and LTU; (**b**) FT-IR and ^1^H-NMR spectra of l-lactide, mPEG, and mPEG-PLA; GPC results of (**c**) LTU and (**d**) mPEG-PLA.

The degradation mechanisms of these polyurethanes can be generally classified as hydrolytic, oxidative, or enzymatic pathways. Hydrolytically degradable polyurethanes are synthesized by the incorporation of soft segments such as polyhydroxy acids and polyesters [35]. Prior to the synthesis of LTU, tyrosine hexyl ester (TH) was synthesized for the protection of the carboxylic acid group of l-tyrosine and confirmed by ^1^H-NMR analysis. Several characteristic ^1^H-NMR chemical shifts at 9.1 ppm and 3.3 ppm were observed for the hydroxyl and amine functionalities, respectively. The ^1^H-NMR spectrum of DTH contained the following characteristic chemical shifts: δ (ppm) = 5.3 (CONH) and 6.6-7.0 (CH_2_ of aromatic ring) with a multiplet. Similarly, as shown in Figure 1a, the ^1^H-NMR spectrum of LTU demonstrated several characteristic peaks: δ (ppm) = 3.3–3.7 (-O-CH_2_-CH_2_-O in PEG), 4.1 (COO-CH_2_-CH_2_), and 2.9 (-NH-CH_2_- in HMDI and C_6_H_4_-CH_2_-CH_2_- in DTH). An important concern in developing biodegradable carriers is the toxicity of their degradation products, which has previously been proven to be negligible with respect to LTU [36]. Taking into consideration their properties of biodegradability and biocompatibility, LTU and LTU NPs were employed in the synthesis and fabrication of nanoparticle gene carriers. LTU—also known as pseudo poly (amino acid)—is designed to be degraded through hydrolysis and enzymatic mechanisms owing to the presence of a peptide bond in its backbone, while PEG is a soft segment macrodiol used to minimize the material toxicity of polyurethanes and to simultaneously increase their flexibility. Prior to the fabrication of LTU NPs, a biocompatible and biodegradable surfactant, the mPEG-PLA block copolymer, was polymerized via the ring-opening polymerization of l-lactide initiated from mPEG (2 kDa) using stannous octoate as a catalyst, and characterized by FT-IR and ^1^H-NMR spectroscopy analyses.

The FT-IR spectra of mPEG-PLA showed the carbonyl group (1750 cm^−1^) of l-lactide and the hydroxyl group (3500 cm^−1^). As seen in Figure 1b, the ^1^H-NMR spectra exhibited the methylene group (-CH_2_) of the repeating unit of mPEG and the methyl group(-CH_3_) of l-lactide at 3.6 and 1.5 ppm, respectively. Additionally, GPC analysis indicates that the LTU polymer possessed a molecular weight of 36,000, along with a polydispersity index of 1.604 (Figure 1c). Biodegradable polymers have a tendency to be extremely bioactive based on their nanoscale organization, making them suitable candidates for use in drug and gene delivery systems [36]. The molecular weight of mPEG-PLA was observed to be approximately 4400 with a polydispersity index of 1.42 (Figure 1d).

The LTU nanoparticles, using the mPEG-PLA diblock copolymer as a biocompatible and biodegradable surfactant, were prepared using the double emulsion solvent evaporation method, which is a common hydrophilic drug and protein encapsulation technique [37]. Although it is commonly accepted that PLA-based drug delivery systems share the long-term release properties of the drugs involved in their biodegradability and biocompatibility, making them one of the most promising polymers for drug delivery system design, these systems have problems such as instability of protein drugs in the process of nanoparticle fabrication or release because the protein is contact with the hydrophobic organic phase and acidic environment owing to the decomposition of the polymer [38]. To solve these issues, we designed a method of delivery of a large amount of genes through rapid release based on swelling caused by osmotic pressure before particles were removed from lysosomes by encapsulating the LPEI-pDNA complex in LTU nanoparticles.

### 2.2. Characterization and Cellular Uptake Ability of FITC-BSA LTU NPs

Two model drugs, FITC-BSA and the pDNA-LPEI complex, were used in this study. In the fabrication process of LTU NPs, these model drugs constitute the primary aqueous phase, while the LTU and mPEG-PLA comprise the organic phase, acting as the matrix and surfactant, respectively. Additionally, 5% PVA aqueous solution was used as the second aqueous phase and a stabilizer for the PEGylation of the LTU NPs. Prior to confirming the cellular uptake ability of LTU NPs, FITC-BSA was initially used as a model drug to verify the encapsulation process in LTU NPs, which could be confirmed by detecting the green fluorescence emitted by FITC-BSA under a fluorescence microscope.

**Figure 2:**
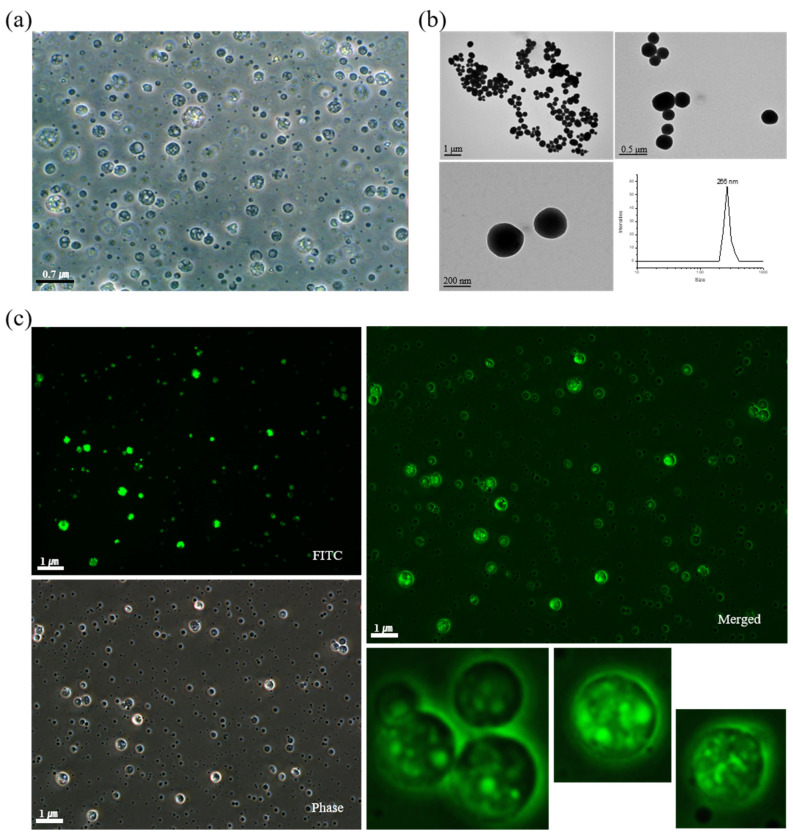
(**a**) Microscopy image of blank LTU nanoparticles; (**b**) TEM and DLS results of FITC-BSA LTU nanoparticles; (**c**) fluorescence microscopy images of FITC-BSA LTU nanoparticles.

**Figure 3:**
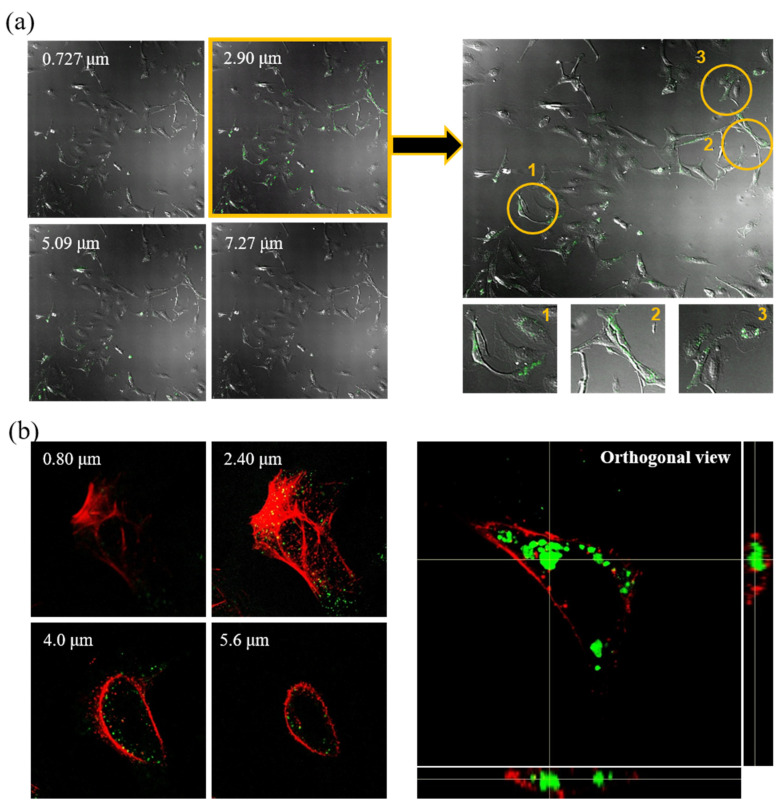
Confocal laser scanning microscopic images of LX2 cells treated with FITC-BSA LTU NPs: (**a**) sliced images with different cell depths and enlarged image at cell depth of 2.90 μm; (**b**) sliced images using rhodamine phalloidin as a dye for actin and its orthogonal view.

The emulsification process was performed using a double emulsion (w/o/w) evaporation method, wherein blank LTU NPs were prepared initially to confirm the properties of double emulsified nanoparticles. As shown in Figure 2a, blank LTU NPs were observed to possess porous spherical shapes with diameters of approximately 400 nm. This size range could prove particularly advantageous for gene and drug delivery through the endocytosis pathway. Due to their sub-micron size, nanoparticles can penetrate deep into tissues and are generally taken up efficiently by cells. In addition, these synthesized polymers exhibit low immunogenicity and are capable of delivering large genes [39,40]. The TEM images and DLS results of the FITC-BSA LTU NPs, shown in Figure 2b, further demonstrated the suitability of their spherical morphology and diameter of 266 nm for endocytosis. Similar findings were also conveyed in the subsequent fluorescence microscopy images, as shown in Figure 2c, which validated the successful encapsulation of FITC-BSA in LTU NPs with a porous spherical shape. Although several attempts have focused on the cellular entry of BSA as a drug carrier when it is fabricated to nanoparticles [41,42], the present research used FITC-BSA as a reference to prove the encapsulation and cell adsorption capacity of LTU nanoparticles because of the fluorescence properties of FITC-BSA. The phase and merge images obtained using fluorescence microscopy similarly indicated a definite porous structure. Moreover, the cellular uptake ability of FITC-BSA LTU NPs was evaluated in LX2 cells wherein the cells were exposed to FITC-BSA LTU NPs overnight and then subjected to confocal microscopy. The number on the confocal microscopy image, shown in Figure 3a, indicates that they were sectioned by a cell depth of 0.727 μm, wherein 0.727 μm and 7.27 μm represent the posterior and anterior surfaces of the LX2 cells, respectively. Green fluorescence was indicative of FITC-BSA LTU NPs, and it was observed that a large number of FITC-BSA NPs were concentrated at cellular depths of 2.90 μm and 5.09 μm. From these sectioned images, we were able to verify that the nanoparticles possessed the appropriate size for successful cellular uptake. Cells marked with yellow circles were enlarged, and it was confirmed that a large amount of LTU NPs encapsulated with FITC-BSA were present within these cells. Similarly, as shown in Figure 3b, the images dyed with actin in red using rhodamine phalloidin—sectioned at 0.8 μm above the cell depth with an orthogonal view—exhibited high intensities of green fluorescence.

Because the ability of biomaterials to adhere to cells is an important factor for confirming biocompatibility, these results demonstrated that LTU NPs have the ability to effectively adsorb to LX2 cells following incubation in a static state for 24 h. In cases of particles smaller than 1 μm, it was previously demonstrated that both clathrin-dependent as well as clathrin- and caveolin-independent endocytosis are utilized to penetrate the cell membrane and form vesicles around the particles, and this endocytosis pathway allows LTU NPs to enter the cells [43].

### 2.3. Characterization of pDNA-LPEI LTU NPs

**Figure 4:**
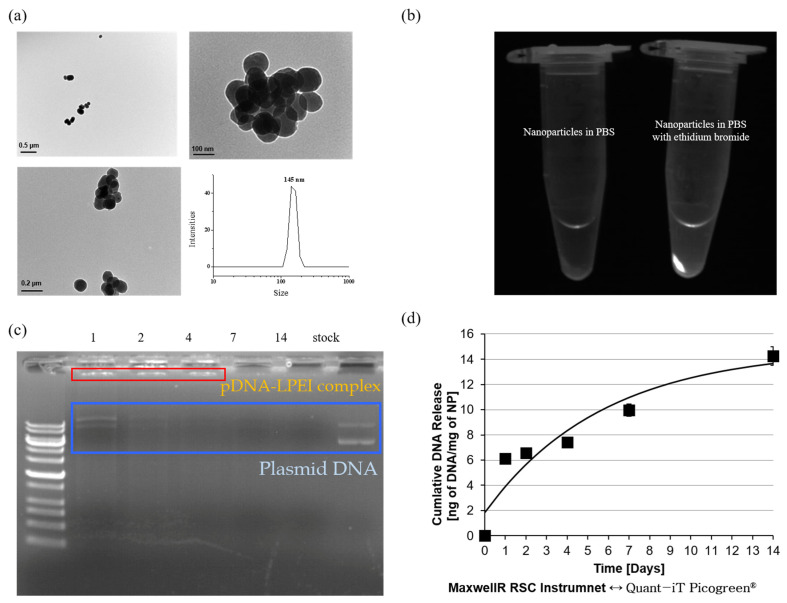
(**a**) TEM and DLS results for pDNA-LPEI LTU nanoparticles; (**b**) pDNA-LPEI LTU nanoparticles with EtBr in PBS for loading confirmation; (**c**) gel electrophoresis image of supernatant obtained from pDNA-LPEI LTU nanoparticles; (**d**) cumulative release of pDNA from pDNA-LPEI LTU nanoparticles with PicoGreen Quantification.

The shape and size of the pDNA-LPEI LTU NPs were evaluated using DLS and TEM, as shown in Figure 4a, which suggested that the diameters of these nanoparticles were approximately 150 nm and they had spherical shapes, and the results obtained from both these experiments were consistent. This size is advantageous for the endocytosis of nanoparticles into cancer cells, which have abnormal walls and membranes compared to normal cells due to their rapid growth. Since it is also important to ensure that DNA is efficiently encapsulated within the LTU NPs, the same was verified using EtBr, which is a molecule that emits a fluorescent signal when combined with DNA. This result indicates that the pDNA-LPEI LTU NPs in PBS did not have any fluorescence, whereas in the case of PBS with EtBr, strong fluorescence was observed. Based on the above comprehensive characterizations, we were able to verify that pDNA was successfully encapsulated within the LTU NPs (Figure 4b). To confirm loading more accurately, an experiment for determining the loading and release studies of the pDNA-LPEI complex was performed by shaking 4.13 mg/mL of the nanoparticles in PBS at 37 °C for 1, 2, 4, 7, and 14 days, then electrophoresis was used with supernatant solution released from the pDNA-LPEI LTU NPs. After shaking each day, the solution was centrifuged at 10,000 rpm at 4 °C, and the supernatant liquid that released pDNA or pDNA-LPEI complexes was poured into a fresh microtube, such that the duration of release of the pDNA or pDNA-LPEI complex could be determined.

In the electrophoresis results depicted in Figure 4c, the red box ladder represents the pDNA-LPEI complex, while the blue box indicates the free pDNA. The presence of the pDNA-LPEI complex was confirmed from these results, which indicated that the complex could be released for at least 4 days. Thus, it can be deduced that pDNA-LPEI formed a complex with increased transfection efficiency owing to the proton sponge effect of LPEI. Cumulative amounts of released free pDNA from the pDNA-LPEI complex demonstrated consistent sustained release behavior, indicating that LTU NPs can be used as a carrier for gene and drug delivery systems. This implies that increased rates of transfection efficiency could be obtained from the complex with LPEI for at least 4 days, thereby aiding in efficient gene therapy. Although PEI exhibits strong cytotoxicity due to the presence of several amine groups, it has been widely used for gene therapy owing to its proton sponge effect, which increases gene delivery efficiency by regulating the osmotic pressure in cells [32]. Following endosome formation by cellular transfusion of the nanoparticles, the unpacking of the complex allows for diffusion of the pDNA into the nucleus. Branched and dendrimer PEIs were not selected in this study due to their high concentration of amine groups, which impart strong cytotoxicity. To conduct further examination of the cumulative release behavior, a Quant-iT PicoGreen assay was performed, wherein the fluorochrome has an excitation maximum at 480 nm and an emission peak at 520 nm, as indicated by the manufacturer, and it selectively binds to dsDNA. When combined with dsDNA, PicoGreen has a very high fluorescence intensity, in addition to possessing a very stable photobleaching effect that facilitates extended exposure times and assay flexibility [44]. The PicoGreen assay employed in this study determined the DNA release behavior of DNA-LPEI LTU NPs with the use of the Maxwell^®^ RSC Instrument, which features automated DNA or RNA extraction with integrated quantification. As shown in Figure 4d, the release behavior of DNA by polymer degradation, as demonstrated by our results, showed a sustained release profile, indicating that LTU NPs are optimal materials for use in gene and drug delivery systems.

### 2.4. Transfection Efficiency of pDNA-LPEI LTU NPs

**Figure 5:**
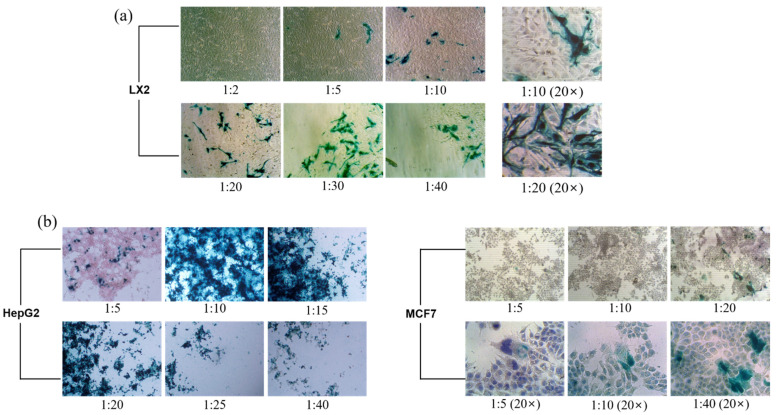
X-gal assay transfection images of (**a**) LX2 cells, and (**b**) HepG2 and MCF7 cells treated with pDNA-LPEI LTU NPs.

Individual infected cells or syncytia were observed in situ with a light microscope owing to their blue color after incubation with X-gal [45]. As reported elsewhere, the transfection efficiency of pDNA-LPEI released from LTU NPs and LPEI toxicity were evaluated in LX2, HepG2, and MCF7 cells using the X-gal assay [46]. The DNA encoded with β-galactosidase used in this assay is capable of emitting blue light upon interacting with X-gal; hence, this procedure is called the X-gal assay, and was applied in this study to elucidate the transfection efficiency of the DNA-LPEI complex. Furthermore, to investigate the cytotoxicity of LPEI, the pDNA-LPEI complex was applied to cells per 200 ng of DNA and the LPEI ratio was adjusted to varying concentrations of 1:2, 1:5, 1:10, 1:20, 1:30, and 1:40, followed by incubation of the cells for 3 days. The transfection results demonstrated the differences in transfection efficiency with varying intensities of blue color, and are displayed in Figure 5a for the LX2 cell as a normal cell, and in Figure 5b for HepG2 and MCF7 cells as cancer cells. These results show that there was a high cell viability when the concentration of LPEI was low, and indicate low cytotoxicity of LTU nanoparticles. In addition, we could confirm that higher concentrations of LPEI caused more cell death due to the increased toxicity of the amine group. In the resulting transfection images, blue color indicates successful DNA transfection after the cellular uptake of pDNA-LPEI LTU nanoparticles, and it was confirmed that the proton sponge effect of LPEI induced the rapid release and increased delivery efficiency of genetic materials via the rapid burst of LTU NPs caused by osmotic swelling. Moreover, we observed that the number of cells with blue color was directly proportional to the concentration of LPEI, as the color was considerably darkened. Furthermore, before proceeding with the transfection evaluation of HepG2 and MCF7 cells as cancer cells, we predicted that more transfections would occur in cancer cells than in normal LX2 cells due to the abnormal structure of the former. Consequently, as shown in Figure 5b, the results obtained for cancer cells exhibited more transfection efficiency than those for the LX2 cells, as expected. This phenomenon may also be responsible for the increase in transfection efficiency due to the hydrogen sponge effect of LPEI.

**Figure 6:**
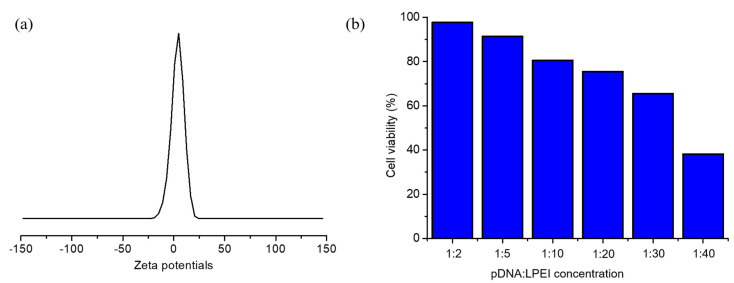
(**a**) Zeta potential result of pDNA-LPEI LTU NPs with a 1:5 ratio of pDNA:LPEI, and (**b**) cell viability of LX2 after X-gal assay transfection investigation using pDNA-LPEI LTU NPs with various LPEI concentrations.

Theoretically, it is commonly accepted that the efficiency of pDNA gene delivery could be enhanced more in cancer cells due to the EPR effect caused by the irregular structures of the widened blood vessel walls in cancer cells, and the electrostatic attraction between the negative nature of cancer cells and the positive charges of pDNA-LPEI NPs [47,48]. The zeta potential result of pDNA-LPEI LTU NPs with 1:5 ratios showed approximately 4 mv, which constitutes supporting evidence that LTU NPs could encapsulate the polyplex formation of pDNA with LPEI (Figure 6a). In in vitro cytotoxicities, the enhanced electrostatic interaction would be considered as a major factor demonstrating increased transfection efficiency. Although treatment with a concentration ratio of 1:5 led to a low degree of transfection in HepG2 cells, treatment with a 1:10 ratio was markedly more successful, due to the significant changes in blue precipitates from 1:5 to 1:10, resulting in much higher transfection efficiency. Additionally, ratios above 1:15 led to apoptosis due to the high cytotoxicity of LPEI. In addition, increasing the concentration of LPEI also correlated with an increase in the concentration of amines, thus confirming that at a high concentration, a large empty space could be observed due to the increased apoptosis of the cells resulting in widened gaps between them. As shown in Figure 6b, these results can also be confirmed by the quantitative analysis of cell viability based on the ImageJ software method, which indicates that the cell viabilities of LX2 decreased from 97.82 to 38.29% with the increasing of the LPEI concentration. Compared to the cells with pDNA and LPEI concentrations of 1:2 and 1:30, the cells treated with a concentration ratio of 1:2 did not exhibit any signs of transfection and 97.82% of LX2 cells remained alive, meaning that LTU nanoparticles are found to be stable in LX2 cells, whereas those treated with 1:30 demonstrated extensive transfection along with dead cells, indicated by the empty regions on the cell fixation device. Based on these results, we were able to determine the optimal concentration ratio of LPEI to be 1:10, which corresponds to the required ratio for the optimal transfection with proper cytotoxicity in HepG2. Although the transfection evaluation in MCF7 cells seemed to yield relatively inaccurate results compared to HepG2 cells, similar transfection profiles were also found depending on the concentration of LPEI. Moreover, in vitro evaluations of anticancer activities and cytotoxicities in common stellate and cancer cells yielded successful results, further demonstrating the potential use of pDNA-LPEI LTU NPs as a novel carrier in gene delivery systems.

## 3. Materials and Methods

### 3.1. Materials

All reagents were purchased from Sigma-Aldrich (Milwaukee, WI, USA), unless stated otherwise. l-lactide and *N*-(-3-dimethylaminopropyl)-N’-ethylcarbodiimide hydrochloride (EDC-HCl), and methoxy-polyethylene glycol (mPEG, Mw ~2000), were purchased from Tokyo Chemical Industry (TCI) (Tokyo, Japan). Dimethylformamide (DMF) was purified by distillation over CaH_2_, while polyethylene glycol was dried prior to its use as a soft segment in LTU. Thionyl chloride was obtained from Daejung Chemical & Metal (Siheung, Korea). pDNA encoding β-galactosidase and 5-bromo-4-chloro-3-indolyl-beta-galactopyranoside (X-gal) and ethidium bromide (EtBr) were purchased from Thermo Fisher Scientific (Waltham, MA, USA). Human hepatic stellate (LX2), breast cancer (MCF7) and liver cancer (HepG2) cell lines were obtained from the Korean Cell Line Bank, Seoul National University (Seoul, Korea). LX2 cells were grown in Dulbecco’s modified Eagle medium (DMEM) supplemented with 2% fetal bovine serum (FBS), whereas HepG2 cells were grown in DMEM supplemented with 10% FBS and 0.5% antibiotic mixture (penicillin-streptomycin). Both the cell lines were incubated at 37 °C and 5% CO_2_.

### 3.2. Characterization

The chemical structures of all synthesized materials were characterized using nuclear magnetic resonance spectroscopy (^1^H NMR; FT-300 MHz Gemini 2000 spectrophotometer, Varian, Palo Alto, CA, USA). The size of the nanoparticles was characterized using dynamic light scattering (DLS; Zetasizer Nano-S90 equipped with a 633 nm He-Ne laser, Malvern Panalytical Korea, Seongnam, Korea) by dispersing the nanoparticles in water. The nanoparticles were also examined using transmission electron microscopy (TEM; JEM-1400, JEOL, Peabody, MA, USA). Furthermore, the double-emulsion structures of the nanoparticles were confirmed using fluorescence microscopy (MX-6300, Meiji Techno, Saitama, Japan). Molecular weight and polydispersity were determined by gel permeation chromatography (GPC), conducted with a Waters 1515 pump and Waters 2414 differential refractometer using Waters columns (Styrogel HR2, HR4, HR5E) in DMF as an eluent at 35 °C and at a flow rate of 1 mL/min. Linear poly(methyl methacrylate) standards were used for calibration. The cellular uptake abilities of the LTU NPs were confirmed by confocal laser scanning microscopy (LSM510NL0, Zeiss, Oberkochen, Germany) with 488 nm excitation for FITC-BSA-encapsulated LTU NPs and 548 nm excitation for rhodamine phalloidin.

### 3.3. Synthesis of LTU

**Figure 7:**
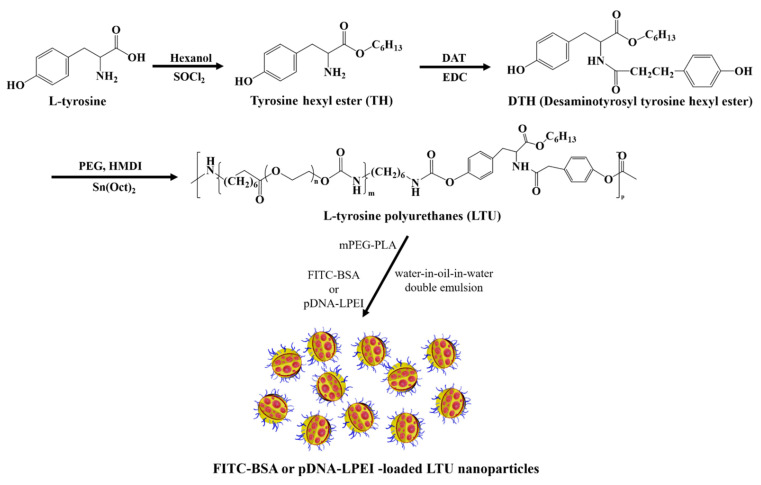
Synthesis of l-tyrosine polyurethane (LTU) and its fabrication of nanoparticles.

The desaminotyrosyl tyrosine hexyl ester (DTH) monomer was synthesized according to the protocol developed by Sen Gupta and Lopina with minor modification [49,50]. Briefly, in the synthesis of LTU, a solution of PEG (0.002 mol) in 40 mL of DMF was added to a mixture of HMDI (0.004 mol) and stannous octoate under a nitrogen atmosphere. The reaction solution was subsequently refluxed at 110 °C for 3 h and cooled down to room temperature with continuous stirring for 10 min. After DTH (0.002 mol) was added to the above reaction solution, the mixture was allowed to react for an additional 15 h at 70 °C. Upon completion of the reaction, LTU precipitated from NaCl aqueous solution was collected using centrifugation and washed with distilled water (Figure 7).

### 3.4. Synthesis of mPEG-PLA

To a solution of mPEG (0.9 mmol) and l-lactide (18.8 mmol) in 120 mL of dried toluene, stannous octoate (100 mg in 100 mg/mL toluene) was added. The reaction mixture was heated to 130 °C under a nitrogen atmosphere. After the reaction was continued for 12 h, the reaction mixture was cooled to room temperature and toluene was completely removed under reduced pressure. The resulting white viscous residues were dissolved in 9 mL of dichloromethane and precipitated into 100 mL of cold ether. The precipitate was collected and dried under vacuum at room temperature for 2 days to obtain mPEG-b-PLA as a white solid [11,38].

### 3.5. Fabrication of FITC-BSA LTU Nanoparticles (NPs)

FITC-BSA LTU NPs were prepared from LTU, mPEG-PLA (with PEG2000), FITC-BSA, and LPEI using the double emulsion (w/o/w) and solvent evaporation techniques [11,26]. Briefly, the gene carrier nanoparticles were fabricated using an initial water-in-oil emulsion of 291 mg of LTU, 3.0 mg of mPEG-PLA dissolved in 10.0 mL of chloroform, and 3.0 mg of LPEI mixed with 3.0 mg of FITC-BSA in 1 mL of autoclaved distilled-deionized water (Table 1). This water-in-oil emulsion was stirred for 1 min at 2000 rpm using a mechanical stirrer (Misung Scientific Lab-Stirrer brushless DC motor BL1020D). The subsequent water-in-oil-in-water emulsion comprising 100 mL of 5% PVA aqueous solution was added to the flask and stirred for 3 min at 1600 rpm. The remaining chloroform was evaporated for 12 h in a beaker equipped with a loose foil cover while being simultaneously stirred and vented. Thereafter, the nanoparticles were collected and washed with D_2_O via centrifugation at 10,000 rpm for 20 min, lyophilized for 72 h (Labconco Freezone 2.5), and finally stored under vacuum. Blank nanoparticles were also fabricated using the same procedure described above without baggage (such as FITC-BSA) in water phase during the water-in-oil emulsion step.

### 3.6. Preparation of pDNA-LPEI LTU NPs

In a similar procedure, pDNA-LPEI LTU NPs were also prepared from LTU, mPEG-PLA (with PEG2000), LPEI, and pDNA using double emulsion (w/o/w) and solvent evaporation techniques. Briefly, the gene carrier nanoparticles were fabricated using an initial water-in-oil emulsion of 194 mg of LTU, 2.0 mg of mPEG-PLA dissolved in 10.0 mL of chloroform, and 6.0 mg of pDNA-LPEI dissolved in 1 mL of autoclaved distilled-deionized water (Table 2). The subsequent steps followed the same procedure as described in the preceding subsection.

### 3.7. Cellular Uptake of FITC-BSA LTU NPs

To investigate the cellular uptake of the nanoparticles, LX2 cells were seeded onto German glass coverslips at a density of 1.5 × 104 cells/cm^2^ and cultured overnight. The FITC-BSA nanoparticles were subsequently suspended in cell culture medium at a concentration of 0.1 mg/mL and exposed to LX2 cells for 24 h, following which the cells were fixed with 0.1% formaldehyde solution in PBS (pH 7.4). As an optional procedure, the fixed cells were stained with rhodamine phalloidin (Thermo Fisher Scientific, WLM, MA, USA). The coverslips were mounted with an anti-fade reagent (Vector Scientific, Golden, CO, USA) and imaged using confocal microscopy (Zeiss, LSM510NL0, Oberkochen, Germany).

### 3.8. Loading Confirmation and Release Behavior Study

Ethidium bromide (EtBr) was used to confirm the loading of pDNA-LPEI [51]. To this end, DNA-LPEI LTU NPs were added separately to freshly prepared PBS (10×) and PBS containing EtBr. Electrophoresis was carried out using the solution obtained after incubating 4.13 mg/mL of the DNA-LPEI LTU NPs in a shaking water bath at 37 °C for 1, 2, 4, 7, and 14 days. The dispersed nanoparticles were subsequently centrifuged at 10,000 rpm at 4 °C for 20 min, and the supernatant liquid containing the released DNA and DNA-LPEI complex was collected to determine the release behavior. A Quant-iT PicoGreen DNA assay kit was used to characterize and quantify the release profile of pDNA from LPEI [52]. After incubating for 1, 2, 4, 7, and 14 days, the collected supernatant was quantified using fluorometry. Since the interaction of pDNA with LPEI decreases the fluorescence of the PicoGreen assay, decomplexation from LPEI allows pDNA to interact with the fluorescence dye, which was consequently used to calculate the amount of pDNA released from the DNA-LPEI LTU NPs.

### 3.9. Transfection of pDNA-LPEI LTU NPs

To qualitatively investigate the transfection efficiency of DNA-LPEI LTU NPs, human hepatic stellate (LX2), hepatic cancer (HepG2), and breast cancer (MCF7) cells were subjected to an X-gal assay, which was used to determine the percentage of transfected cells. Each cell line was seeded onto 24-well tissue culture plates containing feeding media at a density of 25,000 cells/well and subsequently maintained overnight at 37 °C. On the following day, after the feeding medium was replaced, the DNA-LPEI LTU NPs were suspended in fresh feeding medium and added to the wells of the culture plates. The X-gal assay was carried out according to the instructions from the manufacturer with varying concentrations of LPEI. The cytotoxicities in the LX2 cells were measured using ImageJ software (National Institute of Health, Annapolis, MD, USA) [53,54,55].

## 4. Conclusions

A novel biodegradable and biocompatible LTU was synthesized from PEG, HMDI, and DTH as a biodegradable chain extender, and used to fabricate porous nanoparticles for efficient gene delivery using a double emulsion method. The encapsulation and cellular uptake of FITC-BSA in LTU nanoparticles, characterized by fluorescence microscopy and confocal sliced images, showed that a large amount of FITC-BSA NPs had successfully concentrated within the cells, with diameters of 266 nm, which validated the successful gene delivery efficiency of LTU NPs. Furthermore, the LTU NPs encapsulated with pDNA-LPEI had transfection efficiencies both in cancer cells and LX2 cells, which were evaluated by X-gal assay. Based on the experimental results, LTU NPs were found to be promising in their application as efficient gene delivery systems because of their biodegradable, biocompatible characteristics, and sustained release as well as their non-toxic nature. Future studies will focus on in vitro cytotoxicities including the expression of pDNA and transfection efficiency depending on the type of cell lines such as human serum and cell medium.

## Figures and Tables

**Table 1:** Detailed compositions of LTU nanoparticle formulations (Blank and FITC-BSA LTU nanoparticles) produced using the double emulsion method.

Polymer	Conc. (mg/mL)	Mass (mg)	Vol (mL)	Mass % (*w*/*w*)	Vol % (*v*/*v*)
FITC-BSA LTU nanoparticles					
LTU	32.3	291.0	9.0	97.0	8.0
mPEG-PLA	3.0	3.0	1.0	1.0	0.9
FITC-BSA	3.0	3.0	1.0	1.0	0.9
LPEI	3.0	3.0	1.0	1.0	0.9
5% PVA			100.0		89.2
Total		300.0	112.0		
Blank LTU nanoparticles					
LTU	32.6	294.0	9.0	98.0	8.1
mPEG-PLA	3.0	3.0	1.0	1.0	0.9
LPEI	3.0	3.0	1.0	1.0	0.9
5% PVA			100.0		90.1
total		300.0	111.0		

**Table 2:** Detailed compositions of LTU nanoparticle formulations (pDNA-LPEI nanoparticles) produced using the double emulsion method.

Polymer	Conc. (mg/mL)	Mass (mg)	Vol (mL)	Mass % (*w*/*w*)	Vol % (*v*/*v*)
pDNA-LPEI LTU nanoparticles					
LTU	21.6	194.0	9.0	73.0	8.1
mPEG-PLA	2.0	2.0	1.0	6.8	0.9
pDNA-LPEI	6.0	6.0	1.0	20.3	0.9
5% PVA			100.0		90.1
Total		202.0	111.0		

## Data Availability

Data are contained within the article.
